# Supplementary material for: Enhanced Targeted Delivery of Minocycline via Transferrin Conjugated Albumin Nanoparticle Improves Neuroprotection in a Blast Traumatic Brain Injury Model
Source: Brain Sci. 2023 Feb 25;13(3):402. doi: 10.3390/brainsci13030402 (PMC10046830; doi:10.3390/brainsci13030402)
Supplement: Supplementary file 1 [file brainsci-13-00402-s001.zip › brainsci-2205250-supplementary.pdf]

Supplementary file

Experiments

1. **Optimization and validation of HPLC settings and acquisition conditions**

Prior to the encapsulation efficiency and drug release study, the HPLC method was optimized and validated on a Thermo Fisher UltiMate 3000 HPLC, equipped with a photodiode array detector. International Conference on Harmonization (ICH) based recommendations were applied to validate the HPLC method [1,2] [1, 2]. RPC18 analytical reverse phase column (125 mm×4 mm, 5 μm), maintained at ±25 °C, was used with isocratic mode. A mixture of acetonitrile- and water mixture, acidified with 0.02% formic acid (25:75 v/v) was used as the mobile phase at a flow rate of 1 mL/min. All the samples were filtered through a 0.2 μm filter, of which 10 μL was automatically injected into and detected at 273 nm wavelength for a duration of 10 minutes. The obtained and the chromatographic data was further analyzed using Chromeleon CDS software. Minocycline, in concentrations ranging from 0-200.0 μg/mL were used to construct a calibration curve. Minocycline was added to supernatant (blank nanoparticle formulation) for the standard samples. The number of theoretical plates and the tailing factor were estimated for the system suitability by using six replicates of standard minocycline solution at the concentration of 80 μg/mL [3,4].

Further, linearity, specificity, precision, accuracy, robustness, limit of detection and quantification were measured as major validation parameters for ideal HPLC measurement [3]. To estimate the specificity of the HPLC method, a comparison was conducted between the chromatograms of the blank nanoparticles' supernatant and that of the minocycline standards. Precision (Intra- and inter-day variations) was determined using standard samples at concentration (40, 80, and 100 μg/mL) and expressed in terms of the relative standard deviation (% RSD). To determine the accuracy of the measurement, standard samples of known concentration (40, 80, and 100 μg/mL) were compared to the calculated percent recovery of minocycline. By changing the flow rate (0.25, 0.5, and 0.75 mL/min) and ratio (acetonitrile and water; 40:60, 50:50, and 60:40 v/v) of mobile phase, robustness of the HPLC method analyzed using the standard samples (40 μg/mL, 80 μg/mL and 100 μg/ mL) concentration in triplicate. Any changes in these parameters were assessed by RSD and percent recovery. Further, limit of detection and quantification were also calculated from slope of calibration curve and least standard deviation obtained from chromatogram.

Table S1. Experimental control factors and fixed parameters in the optimization of minocycline loaded albumin nanoparticles. Fixed parameters are stirring rate 1000rpm, rate of anti-solvent addition 1ml/min and cross-linking time. Varying parameters were changing the pH and antisolvent to solvent ratio. Initial optimization was performed with 10% of BSA in 5ml HPLC water and 0.25 % of Minocycline.

**Minocycline loaded albumin nanoparticle size and PDI**	135.4±5nm and 0.275±0.01	232.5±2nm and 0.625±0.16	256.6±8nm and 1±0.3
**BSA and Minocycline (weight ratio)**	10% BSA and 0.25% Minocycline	10% BSA and 0.25% Minocycline	10% BSA and 0.25% Minocycline

**pH**	7.0	8.0	9.0
**RPM**	1000	1000	1000
**Rate of addition**	1ml/min	1ml/min	1 ml/min
**Ethanol to Water**	0.65	0.825	1.13
**Cross linking Time (h)**	24	24	24

2. **Minocycline Stability**

Measuring the stability of the drug molecule is essential as it directly impacts the formulation strategy when designing the drug-loaded nanoparticle. Minocycline was subjected to acid and base mediated degradation to understand the stability of minocycline and specificity of the developed HPLC method. 1 M HCL and 1 M NaOH were added to separate minocycline hydrochloride solution and stored for 1h at RT before HPLC analysis. Similarly, a solution of minocycline and H_2O_2 (30%v/v) was allowed to incubate for 1 h prior to HPLC analysis. Light mediated minocycline degradation consisted of visible light exposure on the drug over a period of 24 h.

Results and discussion

3. HPLC Method Validation

The accurate assessment of these parameters is critical given that the nanoparticle must be able to act as a vehicle to carry and transport the drug and produce sustained release of minocycline at the therapeutic target. To accomplish this, a verifiable quantification method needs to be developed to quantify nanoparticle encapsulation[5]. Initial HPLC studies, in reference to previous minocycline quantification in plasma, used a methanol-water mixture [6]. Mobile phases consisting of methanol and water at various ratios were tested to obtain a symmetric peak. However, a regular and symmetrical minocycline peak was only obtained when using an acetonitrile-water mixture acidified with formic acid 25:75 (v/v), at a flow rate of 1 mL/min, with acquisition parameters of column temperature (25 °C), sample temperature (25 °C), injection volume (10 μL), and wavelength (273 nm). Set to these parameters, minocycline was detected at approximately 1.95 min (Fig. S2). Suitability of the developed method was calculated as T=1.27±0.04 and N=7535.14±58.3 which matched with the specified limits (T < 2; N > 2000). Linearity was evaluated at eight concentration levels (0-160 μg/mL) through the method of least squares, resulting in the regression equation $Y = (0.258 \times X - 0.491)$ and correlation coefficient (R=0.998)(supplementary Figure S1)[3]. Specificity of the method was analyzed using the supernatant of the blank, unloaded nanoparticles diluted in acetonitrile. The chromatogram (Fig. S3) was compared with that of the minocycline sample (Fig. S3) and the minocycline standard (Fig.S3). The chromatogram of the supernatant of the blank nanoparticles displayed an additional peak near the retention time, but its small size implies its negligible effect on the quantitative determination of minocycline in nanoparticles (Fig. S3a). Standard samples were made by spiking the supernatant of blank nanoparticles with minocycline standard solution of varying concentrations and then compared to the corresponding minocycline standard. The recovery of

minocycline was nearly 99.8% which indicates that the nanoparticle component from the supernatant showed no interference in relation to the retention time of our peak of interest.

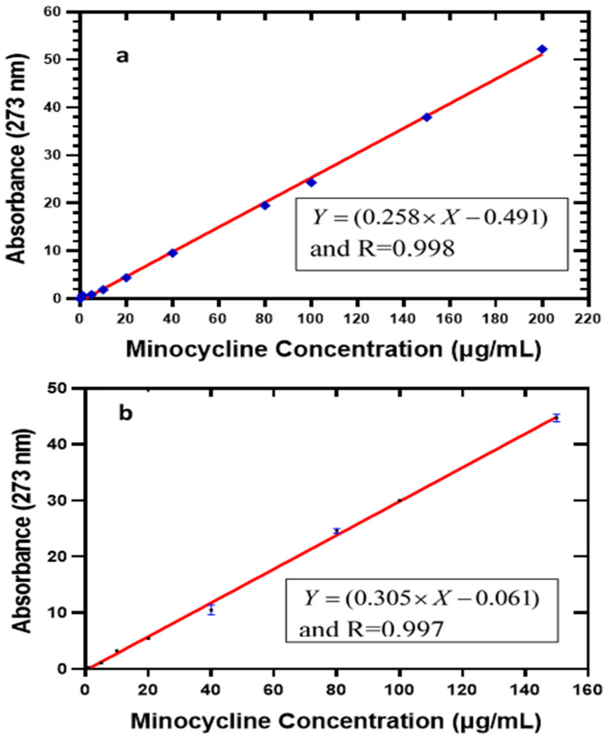

Figure S1. Calibration curve for minocycline standard solution (0-200 µg/mL). a) Calibration curve of minocycline individual run and b) calibration curve of minocycline combined run (n=3).

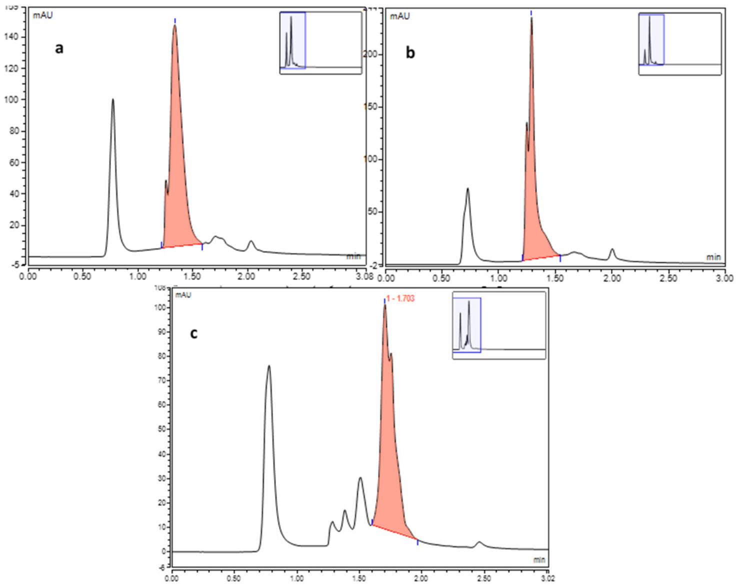

Figure S2. Chromatogram of minocycline analyzed with varying mobile phase (0.02% formic acid in water to acetonitrile) ratio with flow rate of 1ml/min a) 50:50 mobile phase ratio; retention time 1.33 min, b) 50:50 mobile phase ratio; retention time 1.29 min; c) 50:50 mobile phase ratio; retention time 1.78 min.

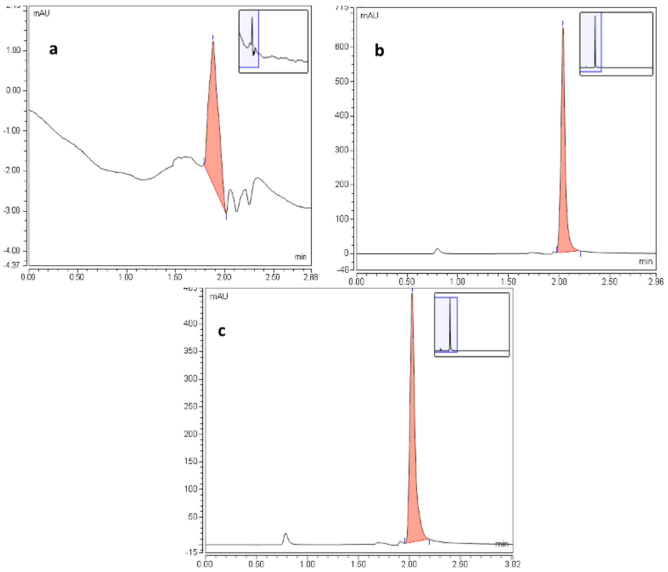

Figure S3. Chromatogram of a) supernatant from blank nanoparticles; b) Minocycline standard solution (150 µg/mL); and c) Supernatant from nanoparticles (minocycline sample, 100 µg/mL).

Table S2. Robustness of the HPLC method to determine minocycline (n=3).

Nominal concentration (µg/mL)	Robustness (% Recovery± % RSD)					
	Acetonitrile (0.2% formic acid) : Water			Flow rate (mL/min)		
	40:60	50:50	60:40	0.25	0.50	0.75
40	37.04±0.4	38.83±0.6	35.46±0.48	169.52±3.1	82.67±1.8	60.89±0.8
80	65.84±0.2	65.65±1.2	47.23±1.4	264.48±8.9	137.70±0.9	92.37±1.5
100	68.98±0.2	69.25±.89	46.16±1.2	272.91±11.7	140.59±0.4	93.07±1.8

Minocycline standard samples (40, 80, and 100 µg/mL) were prepared in triplicate and analyzed on (1) same day (reproducible) and (2) three different days (intermediate precision). The maximum RSD value was mostly below 9.39% indicating the accuracy of the method in Table S2.

Table S3 Precision and accuracy of the HPLC method to determine Minocycline (n=3). *RSD-Relative standard deviation

	Precision (% RSD)	Accuracy (% Recovery)

Nominal concentration (μg/mL)	Intra-day	Inter-day	Intra-day	Inter-day
40	1.08	17.34	111.51	81.37
80	2.84	5.27	112.09	106.82
100	7.06	9.39	109.00	100.74

Table S3 describes the percent recovery of the minocycline standard samples for various flow rates and mobile phase ratios. This method was robust in regard to variations in the volumetric ratio of the mobile phase but displayed sensitivity to flow rate modifications (supplementary Figure S4). In our studies, the minocycline limit of detection and quantification were determined as 125 ng/mL and 250 ng/mL, respectively, which is consistent with previously reported studies in plasma and saliva [3,7].

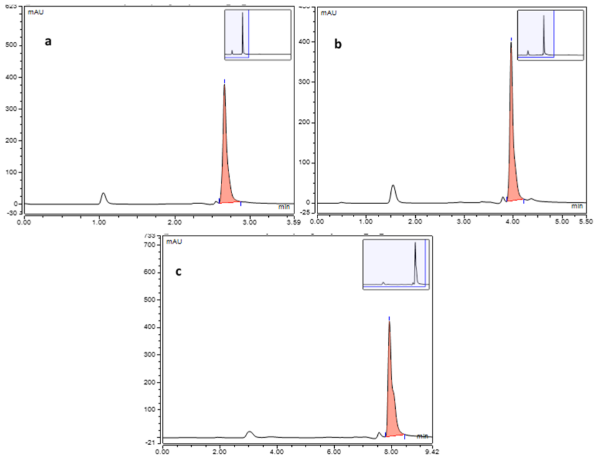

Figure S4. Chromatogram of minocycline analyzed with varying flow rate of mobile phase 0.02% formic acid in water to acetonitrile (75:25) a) 0.75ml/min; retention time 2.6 min, b 0.5ml/min retention time 3.96 min; c) 0.25ml/min; retention time 7.98 min.

As tetracycline degrades under alkaline pH, chelation, and photo-degradation, minocycline degradation was also analyzed. Chromatograms were obtained for minocycline treated with acid, base, peroxide, and visible light and all displayed distinct peaks for minocycline accompanied by minor peaks, with the exception of visible light treatment that contained no additional peaks (Fig.S5d). Specifically, treatment with an acid, base, or H_2O_2 induced minocycline degradation, as displayed in Figs.S5a-c. Acid degraded minocycline contained an additional peak at a retention time of 1.42 min (Fig. S5b). With acid and heating conditions, minocycline readily epimerized at the C-4 position of minocycline (two isomers, 4-epiminocycline) and subsequently the degradant was separated in chromatogram [8,9]. Our results are also consistent with previous reports on minocycline degradation. Similarly, base degraded minocycline exhibited one peak in chromatogram with 1.58 min (Fig. S5a). Minocycline treated with 30% (v/v) H_2O_2 showed completely degraded peak at 1.52 min (Fig. S5c) and no minocycline peak was detected.

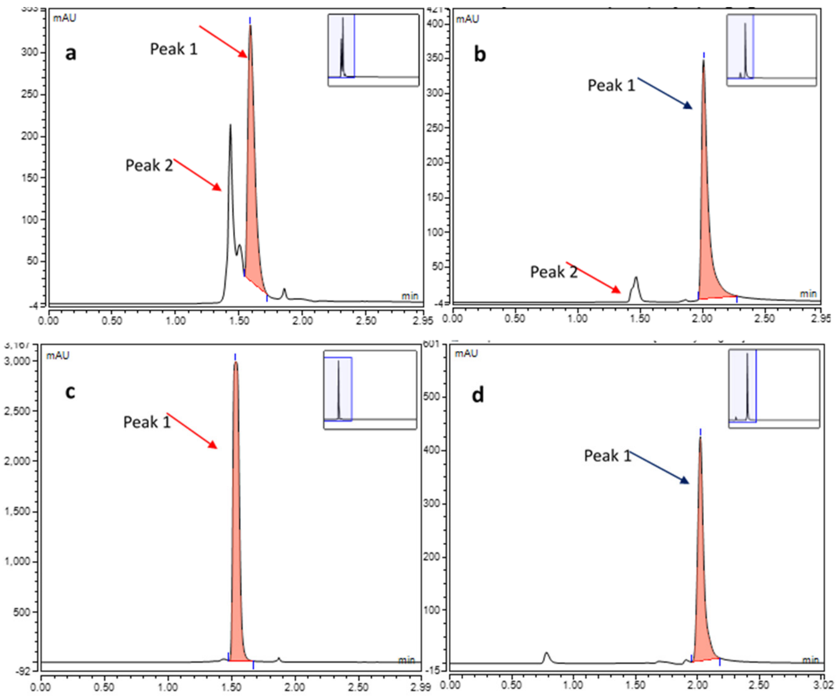

Figure S5. Chromatogram of minocycline treated with a) 1M sodium hydroxide; peak 1 degradant (1.42 min); peak 2 degradant (1.58 min), b) 1 N Hydrochloric acid, peak 1 degradant (1.42 min); peak 2 Minocycline (2.036 min), c) Hydrogen peroxide peak 1 degradant (1.53 min); , and d) light exposure Peak 1 Minocycline (2.15 min).

Table S3 details the percent recovery of minocycline when exposed to various degradation conditions.

Table S4 Results of minocycline forced degradation (n=3).

Exposure Conditions	% Recovery*
UV Light	98.54±0.71
NaOH	74.05±0.4
HCl	88.93±0.32
H_2O_2	undetectable

*The concentration of 100 µg/mL was used

Acid and base exposure led to minor degradation as the minocycline recovery was 88.93 and 74%. Minocycline showed less degradation when spiked in NaCl medium due to its more acidic pH [10]. Jain et al. also reported that this drug is more susceptible to alkaline than acidic degradation which is consistent with our findings. When minocycline was treated with 30% (v/v) H_2O_2, a shift in retention time occurred, rendering percent recovery calculations infeasible and indicating complete minocycline degradation.

Exposure of minocycline to visible light did not hamper percent recovery and resulted in a maximum variation of 2%, indicating stability of the drug. Hence, the developed method is simple, efficient, and sensitive, allowing for the quick analysis of minocycline, and making the method applicable for quantification of minocycline in albumin nanoparticles.

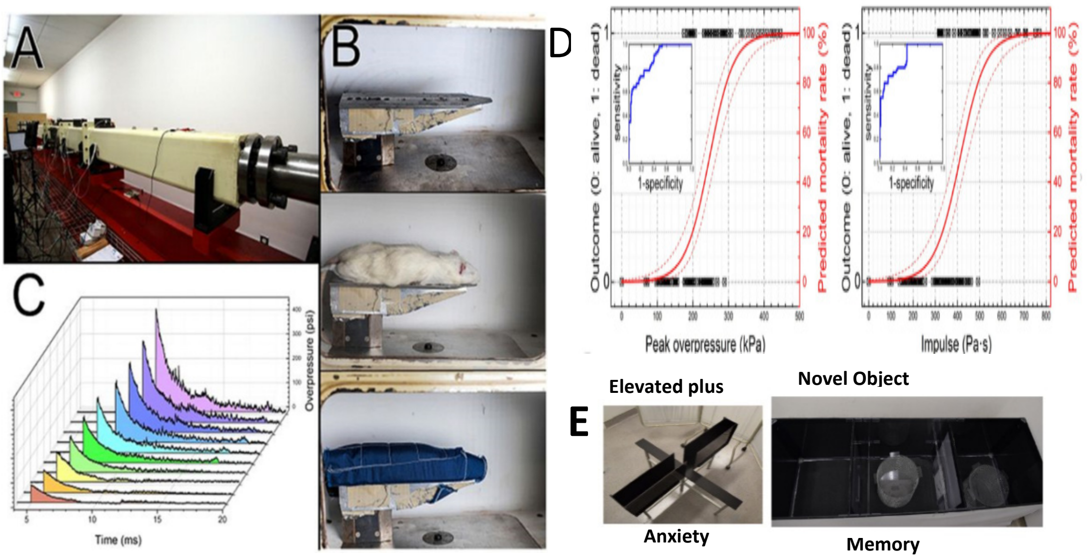

Figure S6. Photo of the 9-inch square cross section, 22 ft long shock tube instrumented with pressure sensors. (B) Aerodynamic rat holder mounted in the test section (top), with rat placed on top (middle) and animal wrapped in a harness to minimize head and body motion during blast exposure (bottom). (C) Representative overpressure profiles as measured in the test section at the location of the animal's head for 10 experimental groups used in our study. (D) The logistic regression dose-response model for rats exposed to single blast with intensity in the range of 60–450 kPa peak overpressure (left) and corresponding impulse (90–780 Pa·s right). Different levels of injury severities are determined based on survival. (E) Setup for behavioral study of anxiety and memory.

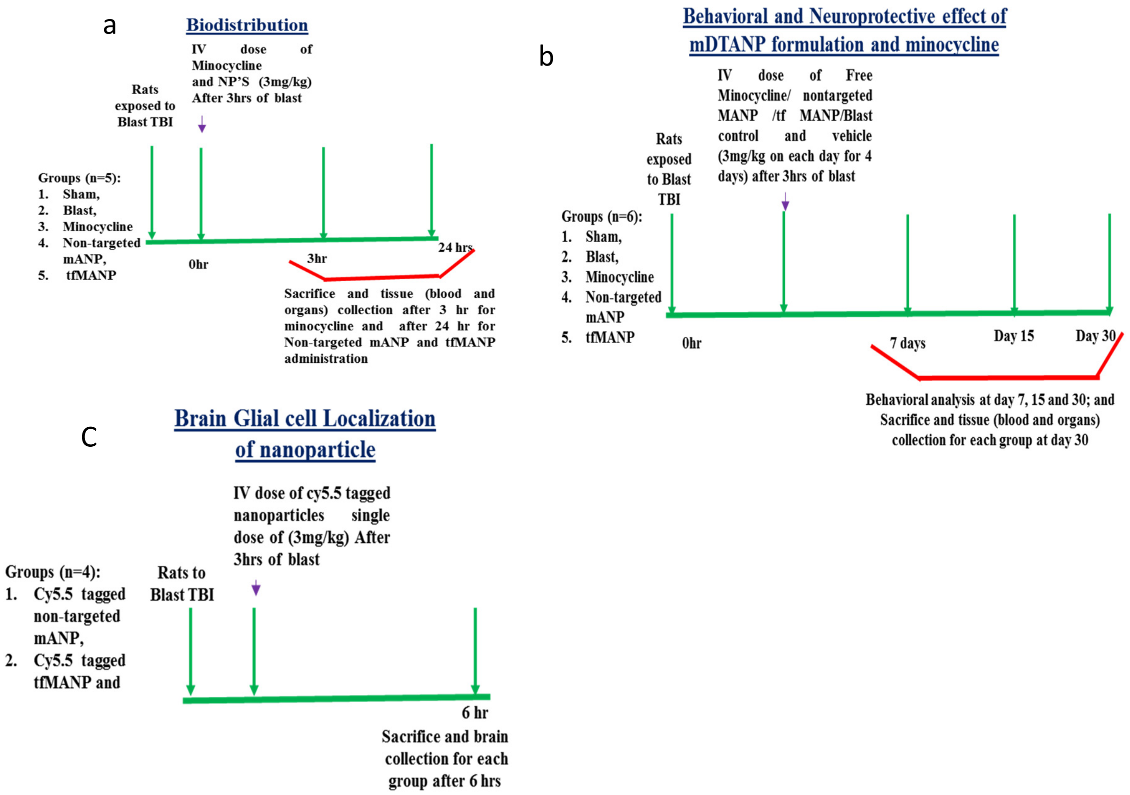

Figure S7. (a) Schematic of biodistribution experiment design; (b) Schematic of Nanoparticle localization experiment design; (c) Schematic of Behavioral and Neuroprotective study.

The laboratory at Center for Injury Biomechanics, Materials and Medicine (CIBM3) has a well-validated blast tube, which is capable of accurately delivering shock waves at different intensities. Animal model of blast TBI to represent diffuse brain injuries using shock tubes is shown in **Fig. S6**. The model is capable of accurately generating shockwaves with different intensities. Additionally, varying blast overpressures (BOPs) from 35 kPa to 350 kPa and using approximately 650 rats the present studies model we have generated dose-response curves which categorized the injury severity from sub-mild, mild, moderate and severe (**Fig. S6**). The BOP we employ in the current study represents moderate injury. Shock Tube, Dose Response Curve and Behavioural Study Setup.

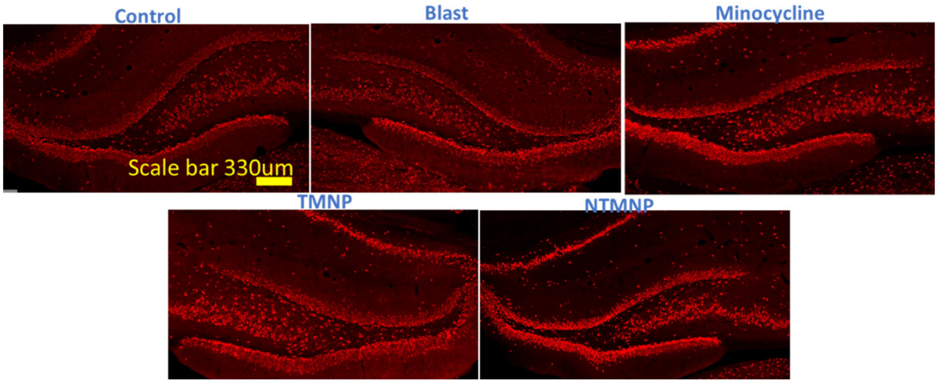

Figure S8. Immunohistochemistry of Free minocycline, TMNP and NTMNP showed neuroprotection against blast induced neuronal cell death.

References

1. Park, E.; Eisen, R.; Kinio, A.; Baker, A.J. Electrophysiological white matter dysfunction and association with neurobehavioral deficits following low-level primary blast trauma. *Neurobiol Dis* **2013**, *52*, 150-159, doi:10.1016/j.nbd.2012.12.002.
2. Jain, D.; Basniwal, P.K. ICH guideline practice: application of validated RP-HPLC-DAD method for determination of tapentadol hydrochloride in dosage form. *Journal of Analytical Science and Technology* **2013**, *4*, 9, doi:10.1186/2093-3371-4-9.
3. de Oliveira, J.K.; Ronik, D.F.V.; Ascari, J.; Mainardes, R.M.; Khalil, N.M. A stability-indicating high performance liquid chromatography method to determine apocynin in nanoparticles. *J Pharm Anal* **2017**, *7*, 129-133, doi:10.1016/j.jpha.2016.08.001.
4. Raval, N.; Mistry, T.; Acharya, N.; Acharya, S. Development of glutathione-conjugated asiatic acid-loaded bovine serum albumin nanoparticles for brain-targeted drug delivery. *J Pharm Pharmacol* **2015**, *67*, 1503-1511, doi:10.1111/jphp.12460.
5. Venkatesan, P.; Puvvada, N.; Dash, R.; Prashanth Kumar, B.N.; Sarkar, D.; Azab, B.; Pathak, A.; Kundu, S.C.; Fisher, P.B.; Mandal, M. The potential of celecoxib-loaded hydroxyapatite-chitosan nanocomposite for the treatment of colon cancer. *Biomaterials* **2011**, *32*, 3794-3806, doi:https://doi.org/10.1016/j.biomaterials.2011.01.027.
6. Wrightson, W.R.; Myers, S.R.; Galandiuk, S. Analysis of minocycline by high-performance liquid chromatography in tissue and serum. *Journal of chromatography. B, Biomedical sciences and applications* **1998**, *706*, 358-361, doi:10.1016/s0378-4347(97)00647-6.
7. Valerie, O.; Audran, M.; Gibert, P.; Bougard, G.; Bressolle, F. High-performance liquid chromatographic assay for minocycline in human plasma and parotid saliva. *Journal of chromatography. B, Biomedical sciences and applications* **2000**, *738*, 357-365, doi:10.1016/S0378-4347(99)00547-2.
8. Nelson, M.; Hillen, W.; Greenwald, R. *Tetracyclines in Biology, Chemistry and Medicine*; 2001.
9. Young, J.E.; Matyska, M.T.; Azad, A.K.; Yoc, S.E.; Pesek, J.J. Separation Differences Among Phenyl Hydride, UDC Cholesterol and Bidentate C8 Stationary Phases for Stability Indicating Methods of Tetracyclines: Journal of Liquid Chromatography & Related Technologies. *J Liq Chromatogr Relat Technol* **2013**, *36*, 926-942, doi:10.1080/10826076.2012.678457.

10. Matos, A.C.; Pinto, R.V.; Bettencourt, A.F. Easy-Assessment of Levofloxacin and Minocycline in Relevant Biomimetic Media by HPLC-UV Analysis. *J Chromatogr Sci* **2017**, *55*, 757-765, doi:10.1093/chromsci/bmx033.
